# Iron metabolism in diabetes-induced Alzheimer’s disease: a focus on insulin resistance in the brain

**DOI:** 10.1007/s10534-018-0134-2

**Published:** 2018-07-24

**Authors:** Ji Yeon Chung, Hyung-Seok Kim, Juhyun Song

**Affiliations:** 1 Department of Neurology, Chosun University School of Medicine and Hospital, Gwangju, 61452 South Korea; 2 Department of Forensic Medicine, Chonnam National University Medical School, Gwangju, 61469 South Korea; 3 Department of Anatomy, Chonnam National University Medical School, Gwangju, 61469 South Korea

**Keywords:** Iron, Diabetes, Alzheimer’s disease (AD), Amyloid beta (Aβ), Insulin resistance

## Abstract

Alzheimer’s disease (AD) is characterized by an excessive accumulation of toxic amyloid beta (Aβ) plaques and memory dysfunction. The onset of AD is influenced by age, genetic background, and impaired glucose metabolism in the brain. Several studies have demonstrated that diabetes involving insulin resistance and glucose tolerance could lead to AD, ultimately resulting in cognitive dysfunction. Even though the relationship between diabetes and AD was indicated by significant evidences, the critical mechanisms and metabolic alterations in diabetes induced AD are not clear until now. Recently, iron metabolism has been shown to play multiple roles in the central nervous system (CNS). Iron deficiency and overload are associated with neurodegenerative diseases. Iron binds to Aβ and subsequently regulates Aβ toxicity in the CNS. In addition, previous studies have shown that iron is involved in the aggravation of insulin resistance. Considering these effects of iron metabolism in CNS, we expect that iron metabolism may play crucial roles in diabetic AD brain. Thus, we review the recent evidence regarding the relationship between diabetes-induced AD and iron metabolism.

## Introduction

Iron contributes to the transportation of oxygen and regulation of cell growth, electron transport and DNA synthesis (Finch 1994; Jehn et al. 2004). Impaired iron homeostasis could result in the excessive production of reactive oxygen species (ROS) and apoptosis (Apostolakis and Kypraiou 2017). In addition, the accumulation of iron contributes to protein misfolding and aggregation, which can lead to multiple diseases (Uversky et al. 2001). Iron gradually accumulates in the brain with age, a process normally associated with changes in iron metabolism (Zecca et al. 2004). The reduction of iron overload using iron chelation therapy has been shown to alter glycemic control in individuals with type 2 diabetes (T2DM) (Swaminathan et al. 2007). One cross-sectional study reported a negative correlation between serum ferritin levels (known as the critical regulator in iron transport and strorage) (Leitner and Connor 2012) and insulin sensitivity (Fernandez-Real et al. 2007). In the central nervous system (CNS), transferrin accounts for approximately 0.4% of the total protein in the brain (Leitner and Connor 2012) and is observed predominantly in white matter (Gebril et al. 2011). Previous in vivo studies have shown that oral administration of iron during brain development triggers memory deficits and induces brain damage in rats (de Lima et al. 2005; Schroder et al. 2001). Current studies have reported that brain iron deposits aggravate cognitive decline in a neurodegenerative disease model (Daugherty and Raz 2015) and higher levels of brain iron deposit in gray matter triggers cognitive dysfunction (Rodrigue et al. 2013). In individuals with mild cognitive impairment (MCI) and Alzheimer’s disease (AD), increased iron levels have been observed in the cortex (Smith et al. 2010). Other research has demonstrated that improvement in iron metabolism may prevent amyloid beta (Aβ) aggregation and ultimately enhance cognition (Adlard et al. 2008). Iron can bind to Aβ (Bousejra-ElGarah et al. 2011) and tau protein (Lei et al. 2017) in the brain. The binding of iron with Aβ induces the aggregation of Aβ and tau hyperphosphorylation (Yamamoto et al. 2002). Consequently, iron could affect the onset and progression of AD in humans. However, the mechanisms of iron accumulation in AD remain unclear. In this paper, we review significant evidence on the influence of iron metabolism on cognitive decline in diabetes-induced AD, and suggest that, similar to the results of an in vivo study that examined the effects of a high fat diet, a common factor between AD and T2DM is the presence of insulin resistance in the brain (Moroz et al. 2008). Taken together, there is a strong relationship between iron metabolism and diabetes-induced AD in terms of the improvement in insulin resistance and the clearance of Aβ in the AD brain. Here, we review recent evidence on the role of iron metabolism in diabetes-induced AD.

## Alzheimer’s disease, impaired glucose metabolism, and insulin resistance

Alzheimer’s disease is the most common neurodegenerative disease and is characterized by cognitive decline, gross atrophy of the cortex and hippocampus, and the aggregation of Aβ and hyperphosphorylated tau (Ramirez-Bermudez 2012; Schubert et al. 2004). It is thought to be various factors affecting the onset and progression of AD, including age, sex, and genetic background (Ramirez-Bermudez 2012; Reitz et al. 2011). Recent research has reported a link between metabolic homeostasis and cognitive decline in obese individuals (Shefer et al. 2013). A large amount of insulin is transported into the brain via movement of cerebrospinal fluid (CSF) across the blood brain barrier (BBB) through saturable and temperature-sensitive mechanisms (Burns et al. 2007; Erol 2008). Insulin acts through its receptors, which are common in the cerebral cortex, hippocampus, cerebellum, and hypothalamus (Hopkins and Williams 1997). Insulin has a crucial role in the brain that relates to neuromodulation, proliferation, and inhibition of neuronal loss (Russo et al. 2005). Case–control studies have demonstrated that insulin resistance (IR) caused by obesity is involved in impaired cognitive function, including both memory function and attention (Maayan et al. 2011), and is associated with an increased risk of dementia (Kodl and Seaquist 2008; Whitmer et al. 2008). In the CNS, insulin influences synaptogenesis and synaptic plasticity and controls glucose metabolism and the secretion of the neurotransmitters involved in cognitive function (Cholerton et al. 2013). Sporadic AD has been reported to involve a state of insulin resistance (Salkovic-Petrisic and Hoyer 2007). IR accelerates neuronal loss by forming advanced glycation end-products (AGE) and ROS (Unoki and Yamagishi 2008). Streptozotocin (STZ)-injected AD models desensitize neuronal IRs and impair brain glucose metabolism, thereby impairing long-term cognitive function in AD patients (Salkovic-Petrisic and Lackovic 2003). Furthermore, AD is characterized by abnormal insulin signaling that results in an insulin-resistant state, increasing Aβ accumulation, tau hyperphosphorylation, and cognitive dysfunction (Talbot et al. 2012). Several studies have demonstrated that insulin deficiency has permissive influence over long-term potentiation (LTP) and memory function (Zhao et al. 2010), increases amyloidosis, and promotes neurobehavioral deficits (Dou et al. 2005; Wang et al. 2010). In addition, patients with T2DM have been shown to have cognitive dysfunction and increased cortical atrophy (Chen et al. 2011; McCrimmon et al. 2012). Impaired glycemic control, cognitive deficits, and a higher risk of AD have been observed in T2DM patients (Hassing et al. 2004; Ronnemaa et al. 2008; Whitmer et al. 2009). A reduced cerebral metabolic rate of glucose has been reported in the AD brain (Small et al. 2000), which is thought to contribute to neurofibrillary tangle formation (Gong et al. 2006).

## Iron in the AD brain

Increase of iron levels has been observed in neurodegenerative diseases (Gozzelino and Arosio 2016; Stankiewicz and Brass 2009) such as AD (Gozzelino and Arosio 2016; Hofer and Perry 2016). In CNS, blood brain barrier (BBB) has been known that it is formed by cerebrovascular endothelial cells (Burdo et al. 2001). One study demonstrated that iron transport into BBB is related with transferrin receptor mediated endocytosis into brain endothelial cells (Jefferies et al. 1984). Additionally, several studies have reported that transferrin receptor mediated signaling is critical the iron uptake across BBB (Beard et al. 2005; Bradbury 1997; Ke and Qian 2007; Moos et al. 2007). Recent studies reported the positive relationship between accumulation of iron in the brain region such as putamen and shrinkage of brain (Daugherty and Raz 2016). Previous studies demonstrated iron accumulation in special brain region such as basal ganglia has been observed based on MRI evidence (Kruer et al. 2012; Levi and Finazzi 2014). One MRI study has demonstrated that elevated iron level in brain are related with impaired cognitive function in obese humans (Blasco et al. 2014). Some CNS diseases, such as AD (Zecca et al. 2004) and Parkinson’s disease (PD) (Oakley et al. 2007), show a relationship between neuronal loss and disturbance of iron metabolism. In AD brains, the increased accumulation of iron is commonly observed in the cortex and hippocampus, white matter areas affected by disease (Antharam et al. 2012; Raven et al. 2013). Another study demonstrated that excess free iron could generate oxidative stress in brain and also contributes the impaired iron homeostasis in AD brain (Altamura and Muckenthaler 2009). There are two forms of iron: redox-active forms such as ferrous (Fe^2+^ iron), and redox-inactive forms such as ferric (Fe^3+^ iron) (Rival et al. 2009). Ferritin, the body’s major intracellular iron storage protein, is elevated in the AD brain (Quintana et al. 2006) and has been observed near AD plaques (Bishop et al. 2002; Connor et al. 1992). The aggregation state of Aβ_1–42_ occurs during the binding of Fe^2+^ and Fe^3+^ and results in the generation of free radicals by activating the iron redox cycle through the Fenton reaction (Khan et al. 2006; Rival et al. 2009). Current study showed that ferritin is found in the Aβ_1–42_ plaque with other proteins and lipids in AD brain (Summers et al. 2017). Several in vitro studies have shown that the coexistence of iron and Aβ reduces neuronal cell viability (Liu et al. 2011; Wan et al. 2011). Redox-active iron forms such as Fe_3_O_4_ have been observed in the human AD brain (Collingwood et al. 2008) and in APP/PS1 transgenic AD mice (Gallagher et al. 2012). Several studies have concluded that the loss of hippocampal integrity in the brains of AD patients is related to increased levels of ferritin (Raven et al. 2013) and decreased ferroportin levels (Raha et al. 2013). Recent study reported that high ferritin level in cerebrospinal fluid (CSF) accelerates the accumulation of Aβ levels in AD brain (Ayton et al. 2015; Quintana et al. 2006). Iron level is positively related to the neuroinflammation on neurons and microglia in AD brain (Cai and Xiao 2016; Urrutia et al. 2013). Moreover, the FerroPortiN1 (FPN1) as the main cellular iron exporter (Abboud and Haile 2000) regulates the iron deficiency or iron overload and its overexpression has been observed in AD brains (Bandyopadhyay and Rogers 2014; Myhre et al. 2013). Also, there are the important diseases in the relationship between AD and iron overload. Iron overload known as hemochromatosis influences various organs such as the liver, heart, and endocrine glands (Gulati et al. 2014; Pelusi et al. 2016). One study demonstrated that the glucose tolerance and diabetes are closely related with the stage of iron overload (Hatunic et al. 2010). Moreover, based on recent studies, the hemochromatosis is one of risk factors in the AD development (Connor et al. 2001; Lehmann et al. 2012; Mariani et al. 2013; Percy et al. 2014). Also, based on the relationship between iron metabolism and lipoprotein metabolism, APOE2, APOE3, and APOE4 could activate APP transcription and trigger the increase of amyloid beta (Aβ) synthesis (Huang et al. 2017). One study demonstrated that the usage of iron chelating drug could enhance the AD pathogenesis by regulating APP processing (Amit et al. 2017). Especially, APOE4 considered the most important genetic risk factor for AD promotes cerebral Aβ deposition (Hare et al. 2013; Kanekiyo et al. 2014; Verghese et al. 2013). Moreover, iron could be more susceptible to bind with Aβ and APOE under amyloid beta toxicity condition (Peters et al. 2015). Furthermore, one case study demonstrated cognitive decline in association with haemochromatosis (Demarquay et al. 2000). Furthermore, imbalance of mitochondrial dynamics are associated with synaptic dysfunction in neuron and cell death in neurodegenerative diseases (Cho et al. 2010). Several studies have indicated that calcium (Ca^2+^) signals affects mitochondrial functions by controlling the activation of Ca^2+^-mediated proteins (Pennanen et al. 2014). Considering recent evidences, iron overload leads to the increase of intracellular Ca^2+^ and affect mitochondrial function in cultured cardiomyocytes and observed in the patients with iron overload cardiomyopathy (Horackova et al. 2000; Khamseekaew et al. 2016). Collectively, the iron overload in AD brain is the critical issue in many insights and could be broadly handled to find appropriated AD therapeutic solution. Taken together, we suggest that iron’s role in the AD brain may be important in elucidating the exact mechanisms of AD pathogenesis.

## Iron and insulin resistance

Iron is known to be a crucial regulator of glucose and lipid metabolism (Fernandez-Real and Manco 2014). Several studies demonstrated the strong relationship between ferritin as the standard marker for iron stores and the increase of diabetes risk (Forouhi et al. 2007; Fumeron et al. 2006; Jiang et al. 2004) such as insulin resistance (Cho et al. 2017; Krisai et al. 2016). Iron blocks the inhibition of insulin of glucose production by the liver and also insulin causes the increased ferritin synthesis in cultured glioma cells (Yokomori et al. 1991). The serum level of ferritin has been known to positively correlate with serum glucose (Fernandez-Real et al. 1998). According to clinical studies, iron overload in body has been reported that it is directly related to the development of glucose intolerance, leading to diabetes (Barbieri et al. 2001; Lao et al. 2001). Fleming et al. shown that the important genes of iron metabolism such as transporters DMT1, ferroportin, and MTP1 were changed in diabetes patients compared to normal subjects (Fleming and Sly 2002). Iron deposition in muscle reduces the uptake of glucose (Fernandez-Real and Manco 2014), and iron influences insulin-producing β-cells by increasing the expression of the iron transporter (DMT1) in the pancreas (Koch et al. 2003). One study has suggested that a possible mechanism for the relationship between serum ferritin levels and insulin resistance is linked to chronic inflammation (Shoelson et al. 2006). Therefore, a high level of serum ferritin is associated with an increase in free radicals and has an influence on insulin resistance (Esser et al. 2014; Gonzalez et al. 2006). Insulin resistance triggers the dysregulation of neuronal insulin signaling and ultimately leads to cognitive dysfunction (De Felice and Benedict 2015; Nuzzo et al. 2015). Insulin has been known to facilitate iron overload by redistribution of transferrin receptors to the cell surface (Noetzli et al. 2012). The oxidative stress by increased iron deposition in beta pancreatic and liver cells leads to insulin resistance, higher insulin secretion and glucose dysregulation (Dongiovanni et al. 2008; Fernandez-Real et al. 2002; Noetzli et al. 2012). In the liver, excessive iron interferes with glucose metabolism, by decreasing insulin extraction and impairing insulin signaling (Ferrannini 2000). Ruivard et al. reported that high fat diet could change iron metabolism (Ruivard 2009; Ruivard et al. 2009) and Meli et al. demonstrated that high fat diet fed animals promotes activity of iron regulatory protein 1 in the liver and an increase of TfR1 expression (Meli et al. 2013). Iron overload inhibits hepatic insulin extraction and the synthesis and secretion of insulin in the pancreas (Fernandez-Real and Manco 2014; Robertson and Harmon 2006). Recent studies have also reported that elevation of serum ferritin levels is linked to insulin resistance (IR) (Batchuluun et al. 2014; Chen et al. 2017). Pharm et al. demonstrated that the level of serum ferritin was positively associated with homeostatic model assessment for insulin resistance (HOMA-IR), an index of IR, in men (Pham et al. 2013). The insulin resistance leads to the high permeability of BBB and triggers cognitive decline in diabetic insulin resistance induced mouse model (Blasco et al. 2014; Takechi et al. 2017) and in AD model (Bell and Zlokovic 2009; Zlokovic 2011). In addition, brain iron overload leads to insulin resistance and subsequently cognitive decline in obesity animal and human models (Cholerton et al. 2013; Fernandez-Real and Manco 2014; Lin et al. 2013; Morris et al. 2011; Schroder et al. 2013; Shefer et al. 2013). One study demonstrated that iron deprivation may promote insulin receptor and Glut4 transcription in muscle (Summers et al. 2017). Considering previous trials, we need the further study to understand the accurate cellular mechanisms between insulin resistance and iron metabolism in AD brain. Collectively, iron overload and deficiency are the critical issues in insulin’s action and its association with insulin resistance. Given that insulin resistance could trigger cognitive impairment (Kong et al. 2018; Lamport et al. 2009; Xu et al. 2009), we speculate that the modulation of iron accumulation could improve cognitive function in AD.

## Conclusions

**Fig. 1:**
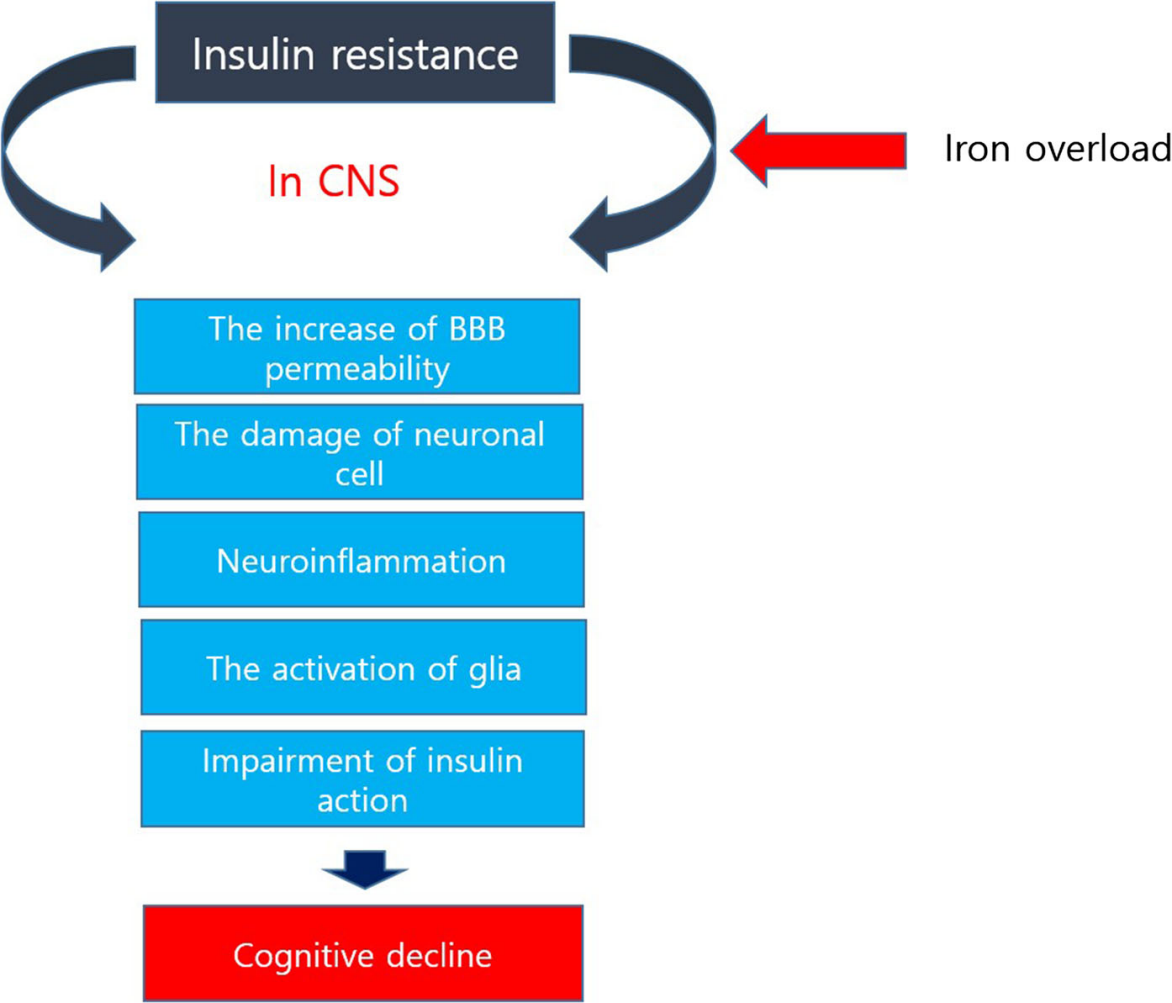
The schematic image between insulin resistance caused by iron overload and AD

Recently, the relationship between diabetes-induced AD has been highlighted because of the common risk factors, such as IR, between AD and T2DM. Here, we reviewed the relationship between iron metabolism and IR in the AD brain (Fig. 1). According to previous studies, iron deficiency could aggravate cognitive dysfunction by way of attention and memory dysfunction and behavioral abnormalities in obese individuals (Jauregui-Lobera 2014; Liang et al. 2014), as well as slower cognitive performance (Lubach and Coe 2008) and perturbation of cognitive development (Bourre 2006). The administration of deferoxamine used in iron overdose recovered motor and sensory nerve conduction velocity and enhanced nerve blood flow in experimental studies (Cameron and Cotter 2001). As well, chronic iron deficiency can trigger cerebral hypoxia and cognitive decline by affecting oxygen transport and storage (Demetri 2001; Petranovic et al. 2008). Although the specific mechanisms regarding the relationship between iron metabolism and cognitive function remain unclear, previous research suggests that iron metabolism is linked to memory function, neuronal survival, and IR in the CNS. Hence, we suggest that the manipulation of iron metabolism in the CNS may be a promising therapeutic approach for treating diabetes-induced AD.
