# Correction to: Iron metabolism in diabetes-induced Alzheimer’s disease: a focus on insulin resistance in the brain

**DOI:** 10.1007/s10534-018-0138-y

**Published:** 2018-09-17

**Authors:** Ji Yeon Chung, Hyung-Seok Kim, Juhyun Song

**Affiliations:** 1 Department of Neurology, Chosun University School of Medicine and Hospital, Gwangju, 61452 South Korea; 2 Department of Forensic Medicine, Chonnam National University Medical School, Gwangju, 61469 South Korea; 3 Department of Anatomy, Chonnam National University Medical School, Gwangju, 61469 South Korea

## Correction to: Biometals (2018) 31:705–714 10.1007/s10534-018-0134-2

Due to a technical error, the copyright line of the above mentioned article was incorrect. The original publication has been corrected.

